# Increased serum vascular endothelial growth factor is associated with acute viral encephalitis in Bangladeshi children

**DOI:** 10.1038/s41598-017-16474-3

**Published:** 2017-11-23

**Authors:** Daisuke Mori, Wahida Khanam, Rahamot Ali Sheikh, S. M. Shahnawaz Bin Tabib, Emi Ikebe, Mohammad Moshaddeque Hossain, Hidekatsu Iha, Kamruddin Ahmed

**Affiliations:** 1 Department of Pathobiology and Medical Diagnostics, Faculty of Medicine and Health Sciences, Universiti Malaysia Sabah, Kota Kinabalu, 88400 Malaysia; 2 Department of Paediatrics, Institute of Child and Mother Health, Dhaka, 1362 Bangladesh; 3 Department of Microbiology, Faculty of Medicine, Oita University, Yufu, 879-5593 Oita Japan; 4 Perdana University Royal College of Surgeons in Ireland School of Medicine, Serdang, 43400 Selangor Malaysia

## Abstract

Encephalitis causes significant global morbidity and mortality. A large number of viruses cause encephalitis, and their geographic and temporal distributions vary. In many encephalitis cases, the virus cannot be detected, even after extensive testing. This is one challenge in management of the encephalitis patient. Since cytokines are pivotal in any form of inflammation and vary according to the nature of the inflammation, we hypothesized cytokine levels would allow us to discriminate between encephalitis caused by viruses and other aetiologies. This pilot study was conducted in a tertiary care hospital in Dhaka, Bangladesh. Viral detection was performed by polymerase chain reaction using patient cerebrospinal fluid. Acute phase reactants and cytokines were detected in patient serum. Of the 29 biomarkers assessed using the Wilcoxon rank-sum test, only vascular endothelial growth factor (VEGF) was significantly higher (P = 0.0015) in viral-positive compared with virus–negative encephalitis patients. The area under the curve (AUC) for VEGF was 0.82 (95% confidence interval: 0.66–0.98). Serum VEGF may discriminate between virus-positive and virus-negative encephalitis. Further study will be needed to confirm these findings.

## Introduction

Acute encephalitis is an inflammation of the brain that requires hospitalization and can lead to severe sequelae, as well as high mortality rates. In industrialized countries, the incidence of encephalitis ranges from 5.23–7.3 cases/100,000 population/year^1,2^ with a mortality rate of 4.6–7.4%^3,4^. In developing countries, the situation is more severe; in India, the incidence is 20.2 cases/100,000/year^5^, and in Vietnam, 30% of hospitalized children with encephalitis die and 25% develop sequelae^6^. In 59–89.9% of encephalitis cases in developing countries, the aetiology is unknown^6–10^. Indeed, this is also true in industrialized countries for 53.3–69.8% cases^4,11–14^. Although >100 viruses are known to cause encephalitis^15^, their distribution varies across regions. In North America, the UK, Australia, and other areas in the West, herpes simplex virus (HSV) followed by varicella-zoster virus (VZV) are primarily responsible for encephalitis^4,11,12,14,15^. However, this profile is changing in some countries. For example, in France, the primary encephalitis-causing virus is enterovirus followed by HSV^16^, and in the US, cases of autoimmune N-methyl-D-aspartate receptor (NMDAR) encephalitis are now outnumbered by those caused by infectious agents^17^. In developing countries, the primary contributing virus also differs. For example, the primary cause is dengue virus followed by HSV type-1 (HSV-1) in Brazil^18^, adenovirus followed by mumps virus in Malawi^19^, Japanese encephalitis virus followed by enterovirus in Vietnam^6^, enterovirus followed by measles virus in India^7^, and adenovirus followed by human bocavirus (HBoV) in Sri Lanka^10^. Therefore, determining the aetiology for viral encephalitis is a challenge that requires comprehensive improvement. New methods such as virus macroarray^20^ and next-generation sequencing^21,22^ are promising approaches; however, these methods do have limitations such as the need for rapid diagnostics in resource-poor settings. Therefore, a simple, practical biomarker is needed to aid in the diagnosis of viral encephalitis.

Cytokines constitute promising and relevant biomarker candidates for detecting viral encephalitis because they are central to any inflammatory process, and encephalitis is a brain parenchyma inflammation caused by infection, post-infection complication, or an autoimmune process^23^. Cytokines levels usually exhibit remarkable association with certain human diseases, which highlights their potential utility as encephalitis biomarkers. Many central nervous system (CNS) infections induce common pro-inflammatory cytokines, including interferon-γ (IFN-γ), tumour necrosis factor (TNF)-α, IFN-γ induced protein (IP)-10, monokine induced by gamma interferon (MIG)^23^. The cytokine spectrum in CNS-infections varied in different studies possibly due to the differences in etiology, age, stage of infection, degree of inflammation, source of sample tested [Cerebrospinal fluid (CSF), serum, plasma etc.], or method used for measuring cytokines (commercial kits versus in-house tests, standards used etc.). The pattern of cytokine elevation may differ depending on the type of viral encephalitis. For example, IFN-γ and TNF-α are elevated in HSV encephalitis, whereas interleukin (IL)-1b is increased in enterovirus encephalitis^23^. However, IFN-γ is not elevated in influenza encephalopathy and human herpesvirus (HHV)-6^23^.

To date, no cytokines that can differentiate between virus-positive and –negative cases have been identified. Such a test would likely have a considerable impact on patient management. Therefore, we hypothesized that differences between certain cytokines may differentiate between encephalitis induced by viruses compared with encephalitis induced by other causes. The purpose of the present pilot study was to identify cytokines and determine the serum levels that would allow us to discriminate between virus-positive and-negative encephalitis cases.

## Results

### Patients

A total of 120 children with acute encephalitis were enrolled in the study. CSF and serum samples were able to be collected from 102 children at the acute stage of infection. However, complete demographic and clinical data only were available for 97 samples; therefore, these were used in the present study. The male to female ratio was 1.7:1. The children ranged in age from 2–114 months, with a mean age of 46.9 months.

### Virus detection in CSF and identification

A total of 18 (18.5%) infection causes were identified (Table 1), while 79 (81.4%) remained unknown. Among the positive samples, 15 (83.3%) were due to a single virus and three (16.7%) were caused by mixed virus infections. HBoV1 was detected in four samples, mumps and adenovirus 41 in two samples each, HHV-1, Epstein-Barr virus, human coxsackievirus B3, human echovirus 2, human parechovirus 3, human adenovirus 40 and human adenovirus 7 in one sample each. In each of the mixed virus infections, HBoV1 was found in combination with human adenovirus 7, 40, or 41.

**Table 1:** Virus identified in the cerebrospinal fluid of patients with encephalitis.

Virus(s) Identified	Number	Percentage
Human bocavirus 1	4	4.1%
Mumps virus	2	2.1%
Human adenovirus 41	2	2.1%
Human herpes virus 1	1	1.0%
Epstein Barr virus	1	1.0%
Coxsackie B virus	1	1.0%
Human parechovirus 3	1	1.0%
Human adenovirus 40	1	1.0%
Echovirus 2	1	1.0%
Human adenovirus 7	1	1.0%
HBoV 1 + HAdV 41	1	1.0%
HBoV 1 + HAdV 7	1	1.0%
HBoV 1 + HAdV 40	1	1.0%
Total detected	18	18.5%

### Analyses of serum cytokines

**Figure 1:**
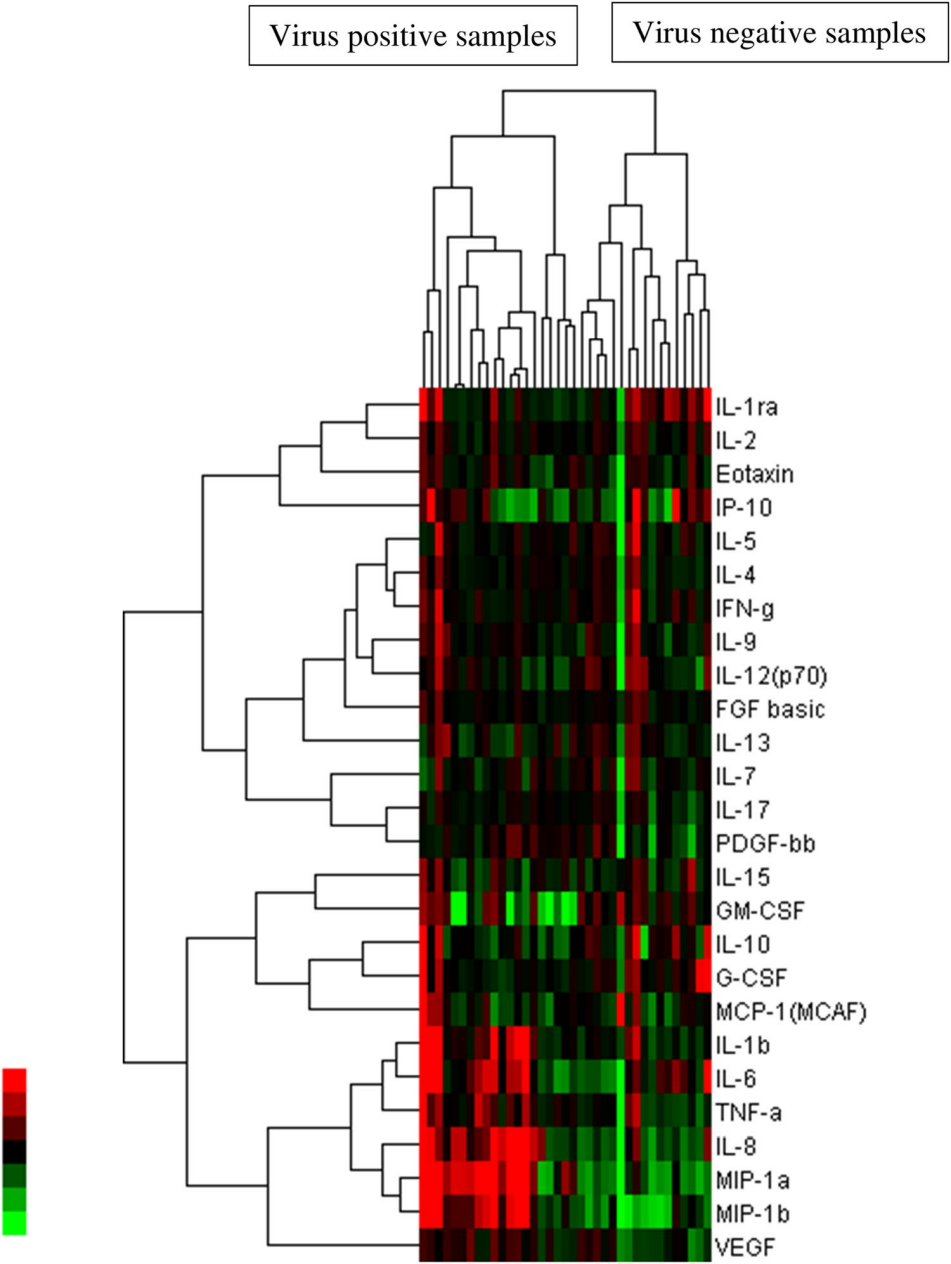
Relative expression of cytokine levels. A heatmap showing relative expression of cytokine levels in virus-positive and virus-negative patients. Cytokine concentrations are expressed in different colours. The values for cytokines are in the rows and virus-status is in the columns. The dendogram describes how variables are related to each other according to cluster analysis. The height where two clusters are merged represents the distances of the two clusters. Color key on the left is showing green as the lowest and red as the highest concentration of cytokine.

The total numbers of samples tested were 93 for C-reactive protein (CRP), 51 for procalcitonin, and 37 each for the other cytokines. The median and the 25th and 75th percentile values of the concentration of each biomarker in virus-positive and virus-negative samples are presented in Table 2. Regulated on activation, normal T cell expressed and secreted (RANTES) was excluded from calculations because levels were the same across samples. We believe sample dilution were not appropriate, disallowing confident measurement within the limit. Hierarchical clustering depicted as a heatmap showed that virus-positive and virus-negative samples separated into two distinct clusters. A dendogram showed that, except for vascular endothelial growth factor (VEGF), all cytokines separated into four clusters (Fig. 1), indicating that the dynamics of only VEGF may have been unrelated to the dynamics of the other cytokines. Furthermore, of the 29 biomarkers assessed using the Wilcoxon rank-sum test, only VEGF was significantly (P = 0.0015) associated with viral infection status, with a higher median in virus-positive samples (304.9 pg/ml) than in virus-negative samples (156.0 pg/ml).

**Table 2:** Comparison of biomarker concentrations in virus-positive vs. virus-negative samples.

Marker	Virus positive			Virus negative			P (Wilcoxon rank-sum test)
	Median	25th percentile	75th percentile	Median	25th percentile	75th percentile	
CRP	0.7	0.4	3.1	0.9	0.2	4.7	0.8597
Procalcitonin	0.6	0.2	1.9	0.2	0.1	2.7	0.3951
Hu IL-1b	5.7	4.6	23.9	4.5	3.4	7.3	0.0605
Hu IL-1ra	247.7	219.3	666.8	297.5	226.4	529.6	0.9746
Hu IL-2	57.7	50.1	84.1	61.1	53.1	76.8	0.5668
Hu IL-4	12.7	11.4	15.4	12.2	10.3	14.8	0.6106
Hu IL-5	18.0	15.7	20.6	21.0	17.5	26.9	0.2865
Hu IL-6	123.4	48.3	255.0	63.9	36.0	132.6	0.1815
Hu IL-7	20.8	16.6	26.4	20.1	16.0	26.1	0.8861
Hu IL-8	232.2	135.8	1337.0	112.3	89.7	501.5	0.1348
Hu IL-9	33.3	29.3	51.5	24.5	22.1	27.6	0.0125
Hu IL-10	30.6	27.7	52.7	27.9	15.1	36.5	0.2032
Hu IL-12(p70)	49.3	44.8	62.5	37.5	26.0	51.3	0.0747
Hu IL-13	13.6	9.5	19.1	14.9	10.8	20.0	0.5776
Hu IL-15	35.5	21.8	39.9	26.8	21.7	41.2	0.5041
Hu IL-17	450.4	392.5	551.7	477.2	407.7	564.3	0.8987
Hu Eotaxin	111.5	80.2	131.0	87.8	62.0	124.8	0.2793
Hu FGF basic	135.2	123.6	155.8	133.9	121.1	152.8	0.5561
Hu G-CSF	95.0	81.1	146.3	99.1	82.4	126.7	0.6675
Hu GM-CSF	81.4	53.5	130.7	58.5	18.6	115.0	0.2264
Hu IFN-γ	182.2	165.0	282.4	183.6	161.7	221.8	0.4171
Hu IP-10	1126.1	631.2	1343.3	963.4	383.1	1845.4	0.5669
Hu MCP-1(MCAF)	94.3	72.6	117.8	106.2	62.5	129.1	0.924
Hu MIP-1a	60.5	11.6	329.2	8.1	4.9	77.1	0.0563
Hu PDGF-bb	5960.7	4709.3	7379.3	5398.5	4228.3	6086.1	0.3730
Hu MIP-1b	823.0	270.8	3311.5	344.6	147.4	814.3	0.1190
Hu TNF-α	128.7	88.6	211.4	101.4	75.7	139.2	0.1522
Hu VEGF	304.9	226.4	337.8	156.0	127.4	227.9	0.0015

**Figure 2:**
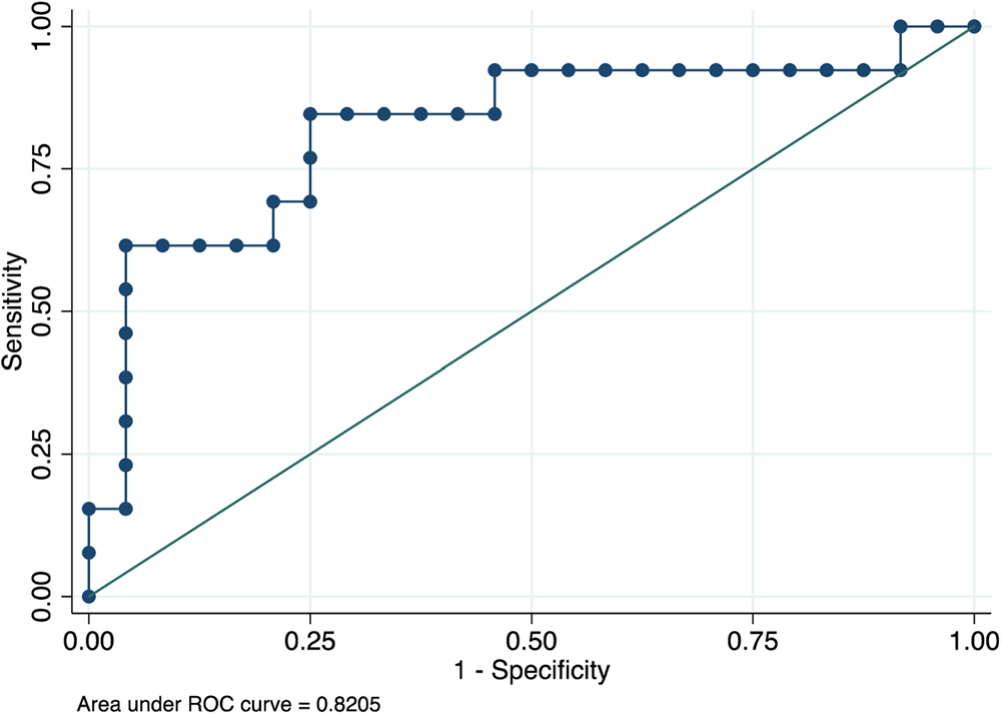
Discriminating abilities of serum Vascular Endothelial Growth Factor (VEGF). The receiver operating characteristics curve showing discriminating abilities of serum VEGF between virus-positive and virus-negative cases of encephalitis.

Receiver operating characteristic (ROC) curve analysis was conducted to obtain the area under the curve (AUC) and validate the ability of VEGF to discriminate between virus-positive and virus-negative samples. The overall AUC for VEGF was 0.82 (Fig. 2), with 95% confidence intervals of 0.66 and 0.98. At the empirically estimated optimal cut-off value (209.8 pg/ml) for this marker, the Youden index was 0.60, sensitivity was 85%, specificity was 75%, and the AUC was 0.80.

## Discussion

Using only clinical features without laboratory support, the specific etiologic agents of encephalitis cannot be determined. Furthermore, the microbiology of encephalitis is not static, and findings from one study period may not be reproducible among the same population in future years^24^. Therefore, a test that can differentiate between virus-positive and virus-negative encephalitis may be valuable at the initial stage of treatment until a specific cause can be identified in the laboratory. Among the currently available CSF biomarkers, pleocytosis, neopterin, and oligoclonal bands are less sensitive and non-specific^23^. MRI abnormalities and evidence of viral infection by PCR or serology may aid in the diagnosis of viral encephalitis^23^, but some of these are time consuming or beyond the reach of clinicians in resource-poor settings. Although acute-phase reactants such as CRP and procalcitonin are increasingly used to identify infections, we could not find significantly higher levels of serum CRP and procalcitonin in virus-positive compared with virus-negative encephalitis cases. Similar results questioning the value of erythrocyte sedimentation rate (ESR) and procalcitonin in meningitis diagnosis have been previously reported^23^.

Many CNS infections induce common pro-inflammatory cytokines such as Th1-related (predominantly IFN-γ, TNF-α, IP-10, MIG) and other cytokines, including interleukin (IL)-1ra, IL-1b, IL-6, and IL-8, are often elevated, suggesting activation of the lymphocytes in the CNS that participate in viral clearance^23^. In the present study, these cytokines were also elevated compared with normal physiological levels (http://www.bio-rad.com/webroot/web/pdf/lsr/literature/Bulletin_6029.pdf), indicating inflammation and host immune reaction to disease. The novel finding of this study is that VEGF serum level is significantly higher in patients with virus-positive compared with virus-negative encephalitis.

It remains unclear whether the VEGF increase in viral encephalitis is induced by viruses as a part of the pathogenesis. However, *in vitro*, VEGF increases the permeability of brain microvascular endothelial cell monolayers by downregulating tight junction proteins, disrupting cell-cell adhesion, and inducing endothelial fenestrations^25^. In addition, it remains unclear whether increased VEGF during viral encephalitis is a host induced protective mechanism for blood-brain barrier disruption because VEGF also plays important roles in wound healing and tissue cytoprotection by stimulating vascular angiogenesis, permeability, and remodelling^26^.

To our knowledge, the utility of serum VEGF in differentiating between virus-positive and virus-negative encephalitis has not been previously reported. However, compared with serum from uninfected controls, VEGF has been reported to be significantly increased in tick-borne encephalitis^27^, which is consistent with our observation. Other evidence indicates that VEGF in CSF significantly increases in bacterial meningitis, including active tubercular meningitis^28,29^.

One limitation of our study was that, due to the insufficient number of samples we were unable to determine the level of cytokines in CSF. Therefore, the dynamics of VEGF in CSF during encephalitis remain unclear. Contrary to our finding of viral encephalitis, serum VEGF is decreased in bacterial meningitis^28^. Another limitation was that we did not use a next-generation sequencer to detect novel viruses. However, we expect that the number of novel viruses among the total cases would have been too small to affect our overall findings^30^.

We identified serum VEGF as a possible biomarker for differentiating between virus-positive and virus-negative encephalitis. This distinction may facilitate patient management by guiding decisions about administering antiviral drugs. Our observation also supports the need to determine the effects of anti-VEGF treatment^28^ in the clinical course and outcome of virus-positive encephalitis. Larger studies are needed to validate the usefulness of serum VEGF concentrations in accurately predicting the presence of viruses in the CSF of encephalitis patients.

## Methods

### Patients

We conducted an observational, non-interventional study of paediatric patients from April 2010 through August 2012 at the Institute of Child and Mother Health (ICMH) Hospital in Matuail, Dhaka, Bangladesh. This tertiary care hospital serves approximately 4.8 million people in its catchment area and has 200 beds, among which, 85 are dedicated to paediatrics. The study was approved by the ICMH ethics committee. All experiments were performed in accordance with relevant guidelines and regulations. Children with encephalitis admitted to the paediatric ward during the study period were enrolled. Encephalitis was defined as presence of fever, convulsion, and unconsciousness with or without signs of meningeal irritation. Samples were collected from children whose guardians provided verbal consent for their participation. Only samples remaining after the completion of routine tests were used for this study.

### Routine laboratory investigations

CSF samples were subjected to macroscopic examination, total and differential white blood cell (WBC) counts, bacterial culture, Gram staining and measurement of protein and glucose levels. Blood was cultured for bacteria and examined for total and differential WBC counts, ESR, and haemoglobin and CRP levels.

### CSF virus and bacteria detection and identification

Genomic DNA and RNA were extracted using a QIAmp viral RNA mini kit (Qiagen Company Ltd., Tokyo, Japan) and Trizol reagent (Invitrogen, Carlsbad, CA, USA) according to the manufacturers’ instructions.

Altogether, we tested for 41 encephalitis-causing viruses using previously published methods. HSV-1, HSV-2, cytomegalovirus, VZV, (Human herpes virus) HHV-6, HHV-7, and HHV-8 were tested using universal HHV primers^9,10^. Dengue, Japanese encephalitis, West Nile, yellow fever and tick-borne encephalitis viruses were tested using universal flavivirus primers^9,10^. Nipah, measles, mumps, parainfluenza, respiratory syncytial and metapneumoviruses were tested using *Paramyxovirinae* and *Pneumovirinae* primers^9,10^. Chikungunya, Sindbis, Semliki forest, Eastern and Western equine encephalitis viruses were tested using generic alphavirus primers^9,10^. Poliovirus, coxsackievirus, and echovirus were tested using human enterovirus-specific primers. Rabies virus, Chandipura virus, rotavirus, astrovirus, HBoV, norovirus, and enteric adenovirus were detected using specific primers^9,10^. Bufavirus^30^, Merkel cell polyomavirus^31^, WU virus^32^, KI polyomavirus^32^, trichodysplasia spinulosa-associated polyomavirus^33^, cardiovirus^34^, human parechovirus^35,36^, and Liao ning virus^37^ were also tested using respective primers. Bacteria were detected via PCR using primers for the 16S rRNA gene^9,10^.

### Nucleotide sequence

Nucleotide sequences of the PCR products were determined to confirm the results and distinguish viral types. The amplicons were sequenced with an ABI Prism 3130 Genetic Analyzer (Applied Biosystems, Foster City, CA, USA) using the BigDye Terminator v3.1 Cycle Sequencing Kit (Applied Biosystems), according to the manufacturer’s instructions.

### Measurements of serum cytokines

Serum levels of human IL-1b, IL-1ra, IL-2, IL-4, IL-5, IL-6, IL-7, IL-8, IL-9, IL-10, IL-12 (p70), IL-13, IL-15, IL-17, eotaxin, fibroblast growth factor basic, granulocyte-colony stimulating factor (G-CSF), granulocyte macrophage-CSF, IFN-γ, chemokine (C-X-C motif) ligand (CXCL)-10 (IP-10), chemokine (C-C motif) ligand (CCL)-2 [Monocyte chemoattractant protein (MCP)-1], CCL-3 [Macrophage inflammatory protein (MIP)-1a, CCL-4 (MIP-1b), platelet derived growth factor, CCL-5 (RANTES), TNF-α, and VEGF in 14 virus-positive and 24 virus-negative patients were measured in duplicate using a multiplex sandwich immunoassay-based protein array system (Bio-Plex Pro Human Cytokine 27-Plex Assay kit; Bio-Rad Laboratories, Hercules, CA, USA) according to the manufacturer’s instructions.

### Cluster analysis and heatmap generation

To reveal cytokine profiles in patients with viral encephalitis, we performed cluster analysis on the virus status of samples (positive or negative) and serum cytokine levels. This analysis employed clustering in two ways based on: (i) serum cytokine level and (ii) presence or absence of any virus in the samples. The results are represented as a heatmap. Data were formatted using Microsoft Excel (Microsoft Corporation, Redmond, Washington, USA). Clustering was done by Cluster 3.0 (http://bonsai.hgc.jp/~mdehoon/software/cluster/software.htm) using hierarchical clustering, and then visualized using Java TreeView (http://jtreeview.sourceforge.net/).

### Statistical analysis

The concentrations of 27 of the 29 biomarkers tested were non-Gaussian distributed. Therefore, we summarized the concentrations as the median and the 25th and 75th percentile values and used the nonparametric Wilcoxon rank-sum test to assess whether the concentration of acute phase reactants and cytokines differed significantly by viral infection status (positive vs. negative). For Wilcoxon rank-sum tests, we considered a difference significant if the obtained P-value was smaller than the Bonferroni-adjusted α of 0.0017.

The ROC curve was used to obtain the AUC in order to evaluate whether each cytokine can be used to make a distinction between the two etiological groups. In addition, for significant candidates, the Youden index was calculated to identify the point of maximum sensitivity and specificity and to describe its potential effectiveness. All statistical analyses were performed using Stata 14.0 (StataCorp LP, College Station, TX, USA).
